# Reduction in adolescent depression after contact with mental health services: a longitudinal cohort study in the UK

**DOI:** 10.1016/S2215-0366(17)30002-0

**Published:** 2017-02

**Authors:** Sharon A S Neufeld, Valerie J Dunn, Peter B Jones, Tim J Croudace, Ian M Goodyer

**Affiliations:** a Department of Psychiatry, University of Cambridge, Cambridge, UK; b School of Nursing and Midwifery and Social Dimensions of Health Institute, University of Dundee, Dundee, UK

## Abstract

**Background:**

Evidence regarding the association between service contact and subsequent mental health in adolescents is scarce, and previous findings are mixed. We aimed to longitudinally assess the extent to which depressive symptoms in adolescents change after contact with mental health services.

**Methods:**

As part of a longitudinal cohort study, between April 28, 2005, and March 17, 2010, we recruited 1238 14-year-old adolescents and their primary caregivers from 18 secondary schools in Cambridgeshire, UK. Participants underwent follow-up assessment at months 18 and 36. Trained researchers assessed the adolescents for current mental disorder using the Schedule for Affective Disorders and Schizophrenia for School-Age Children Present and Lifetime version (K-SADS-PL). Caregivers and adolescents reported contact with mental health services in the year before baseline. Adolescents self-reported depressive symptoms (Mood and Feelings Questionnaire [MFQ]) at each timepoint. We assessed change in MFQ sum scores from baseline contact with mental health services using multilevel mixed-effects regression adjusted for sociodemographic, environmental, individual, and mental health confounders, with multiple imputation of missing data. We used propensity score weighting to balance confounders between treatment (users of mental health services) and control (non-users of mental health services) groups. We implemented an MFQ clinical cutoff following the results of receiver operating characteristic analysis.

**Findings:**

14-year-old adolescents who had contact with mental health services in the past year had a greater decrease in depressive symptoms than those without contact (adjusted coefficient −1·68, 95% CI −3·22 to −0·14; p=0·033). By age 17 years, the odds of reporting clinical depression were more than seven times higher in individuals without contact than in service users who had been similarly depressed at baseline (adjusted odds ratio 7·38, 1·73–31·50; p=0·0069).

**Interpretation:**

Our findings show that contact with mental health services at age 14 years by adolescents with a mental disorder reduced the likelihood of depression by age 17 years. This finding supports the improvement of access to adolescent mental health services.

**Funding:**

Wellcome Trust, National Institute for Health Research.

## Introduction

Many mental disorders emerge during adolescence and continue into adulthood.^1^ In depressive disorders, younger onset is associated with more depressive episodes, longer episode duration, increased comorbidity, suicidality, and admission to hospital.^2^ Among individuals with a diagnosed depressive disorder, adolescents are more likely than adults to delay contact with mental health services, thereby increasing episode duration and risk of recurrence. Clearly, early identification and treatment of mental disorders during adolescence would contribute to reduction and perhaps prevention of adverse sequelae.

Measurement of the treatment gap—the discrepancy between disorder prevalence and proportion treated—is a prerequisite to enable policy makers to prevent such adverse sequelae from arising. To predict service need, a clearly recognised cutoff for mental disorder, such as meeting DSM diagnostic criteria, is desirable. Our review of international studies that report DSM-IV disorder and past-year contact with mental health services for those with a disorder (appendix pp 1, 2), found that 12–25% of adolescents have a mental disorder, of whom only 34–56% access mental health services. Previous surveys^3^, ^4^ in the UK report much higher proportions of contact with mental health services (71% of children or adolescents with a mental disorder); however, unlike most studies, these estimates classify seeking help from a teacher as a mental health service contact. Other studies^5^, ^6^ report 12–19% lower service use rates for anxiety than for depression.

The association between adolescents' contact with mental health services and subsequent mental health remains unclear in community samples, but is vital to clarify if adolescent mental health services are to compete for health-care funding. Findings from studies^7^, ^8^ using broad definitions of mental health problems without a cutoff for service need have shown that use of mental health services had little effect on subsequent mental health problems. However, results are more promising if adolescents are at greater risk of, or already have, a mental disorder. In adolescents who witnessed community violence, use of mental health services reduced depressive symptoms.^9^ Adolescents with fearful spells or panic attacks were more likely to develop diagnosable panic disorder and depression if they had not used mental health services.^10^ Patients treated for emotional disorders at Child and Adolescent Mental Health Services (CAMHS) showed significant improvement compared with controls,^11^ yet this change was not clinically meaningful. Finally, among DSM-diagnosed adolescents, users of specialist mental health services had reduced symptoms compared with those who were untreated, but only if eight or more sessions were attended.^12^ However, none of the studies that showed a positive association between service contact and mental health addressed non-randomisation or attrition. Only one study^9^ adequately addressed confounding variables (ie, those associated with both predictor and outcome, which could bias the association between service use and subsequent mental health), and only one study^10^ showed significant effects that were clinically relevant.

**Research in context**

**Evidence before this study**

In 2015, a task force in the UK noted the paucity of good quality national information regarding Child and Adolescent Mental Health Services (CAMHS) outcomes. To identify previous published work, with no language restrictions, that assessed the association between CAMHS use and subsequent mental health, we searched PubMed (* denotes wildcard) for articles published in the past 16 years (from Jan 1, 2000, to July 5, 2016) for the terms (service* OR help-seek*) AND (psychopatholog* OR mental* OR psychiatric*) AND (observation* OR community OR survey OR cohort OR epidemiolog*) AND (longitudinal[Title] OR prospective[Title] OR change[Title] OR reduc*[Title] OR improve*[Title] OR effectiveness[Title] OR outcome[Title]) AND (adolescen*[Title] OR youth*[Title] OR young*[Title]). We required studies to reflect treatment-as-usual mental health service use, and have a non-service using comparison group. We identified additional papers by checking citations.

We identified six studies that yielded mixed findings regarding the association of service contact with subsequent mental health. Two studies that assessed change in all service users without a clearly recognised cutoff for service need, such as DSM, showed that mental health service use had little effect on subsequent total mental health problems over and above that to be expected from natural remission. The four remaining studies assessed adolescents at greater risk of a mental disorder or those with a DSM diagnosis. These studies showed an improvement in mental health following service contact, but none addressed non-randomisation of service contact or attrition, only one adequately addressed confounding variables, and only one showed significant effects that were clinically relevant. None of these studies were from the UK (three were from the USA and three were from Europe).

**Added value of this study**

To our knowledge, this study is the first of its kind in the UK, and the first to support the association of mental health service contact and the improvement of mental health by late adolescence, while addressing non-randomisation of service contact and attrition. In addition to propensity score weighting (which balances treatment and control groups on confounders, similar to a randomised control trial) to adjust for participants' initial likelihood to access services, and multiple imputation to deal with missing data, we used a clinically relevant cutoff and adjusted for a wide range of time-varying confounding variables. These adjustments give greater confidence than previous studies to the notion that mental health service contact is related to meaningful improvements in subsequent mental health. This study is also the first we are aware of that shows that the association of mental health with previous treatment is attenuated if that treatment was irrespective of service need.

**Implications of all the available evidence**

The spending of the UK National Health Service (NHS) on children's mental health services has fallen by 5·4% in real terms since 2010 (£41 million), despite an increase in demand. The present findings support the positive role played by mental health services in a cohort before these NHS cuts, illustrating to policy makers the validity of increasing the availability of child mental health services to at least 2010 levels. That positive findings became non-significant upon inclusion of all mental health service users irrespective of disorder underscores the importance of clinical assessment when making referral decisions. These findings support training of service referrers (eg, in primary care or schools) in detection of the presenting features of mental disorders, to increase the proportion of referrals of individuals with a clear need who could be more responsive to treatment.

In the present study, we used a longitudinal repeated-measures design on a community ascertained cohort to assess change in adolescent depressive symptoms from ages 14 years to 17 years after contact with mental health services. For the outcome, we used depressive symptoms as a valid identifier of major depressive disorders,^13^, ^14^ which are highly prevalent^5^ and predictive of future morbidity.^2^ To extend this previous work, the design controls for differences in symptoms and background factors among service users and non-users at baseline and over time, in individuals with and without a DSM-defined mental disorder. We hypothesised that self-reported depression scores would be reduced to a greater extent in adolescents who contacted mental health services than in those with no contact, but that these effects would be stronger in the subsample with a clearly defined need for mental health services, based on the presence of a diagnosable mental disorder. We hypothesised that these findings would remain when we addressed attrition, confounding variables, non-randomisation of mental health service contact, and clinical relevance.

## Methods

### Study design and participants

As part of the ROOTS longitudinal cohort study^15^ of mental health, between April 28, 2005, and March 17, 2010, we recruited 1238 adolescents and primary caregivers (1134 [92%] were the biological mother of the adolescent) from 27 secondary schools in Cambridgeshire, UK. 18 secondary schools approached agreed to participate, with letters of invitation sent until the sample size reached a prespecified cutoff of 1000 participants. Of a possible 3762 students, 1238 agreed to participate. Participants were interviewed separately and completed questionnaires at mean ages 14·5 years (timepoint 1 [T1]), 16 years (timepoint 2 [T2]), and 17·5 (timepoint 3 [T3]) years (T1–3 means, SDs 0·3). Written informed consent was obtained from adolescents and caregivers before participation. Cambridgeshire 2 Research Ethics Committee local ethics committee provided ethics approval.

### Procedures

At T1, trained researchers assessed adolescents' mental health status using the Schedule for Affective Disorders and Schizophrenia for School-Age Children–Present and Lifetime version (K-SADS-PL)^16^ to establish DSM-IV^17^ axis 1 diagnoses. Adolescents with a high clinical index (subthreshold for diagnosis, but exhibiting four symptoms and clinical impairment) were classified as diagnosed. Experienced psychiatrists (IMG, PBJ) trained interviewers and conducted consensus meetings regarding all K-SADS assessments. Inter-rater agreement for diagnosis was high (95%). Disagreements were settled by clinical consensus meetings between clinical psychiatry experts.

Mental health service contact was defined as an adolescent's assessment or treatment of a mental health problem by a primary care provider (ie, general practitioner) or a mental health specialist from any sector. Caregiver and adolescent responses were obtained by use of various measures (items in appendix pp 3–5). T1 past-year mental health service contact (no or yes) was generated as an exposure variable, and any mental health services after T1 (no or yes, post-T1–3) as a confounder. Caregivers reported contacts with adolescent mental health services at T1 from a semi-structured interview, with high inter-rater agreement on core indicators (κ=0·7–0·9; Cambridge Early Experiences Interview^18^) and from a self-reported questionnaire at T1 and T3. Adolescents were interviewed at T3 about mental health service contact before K-SADS-PL assessment. Adolescents also reported how often they had seen a doctor or other health professional regarding depressive symptoms in the past month (Kessler's Psychological Distress Scale^19^). We combined adolescent and caregiver responses with either response if one was missing, or with the positive response if sources disagreed (considering differential recall and caregivers potentially unaware of adolescent service use).

A combined variable was derived at T1 that defined participants with current mental disorder (yes or no) and past-year mental health service contact. This variable resulted in four levels: unaffected (no current disorder or past-year service contact), service contact only, disorder only, and disorder and service contact.

We assessed the Mood and Feelings Questionnaire (MFQ)^20^ at T1–3. This 33-item adolescent self-report of current or past 2 weeks' depressive symptoms covers DSM criteria for major depressive disorders. The MFQ has shown prognostic validity in clinic and non-clinic samples,^13^, ^14^ yielding high internal consistency (α=0·92–0·94) in the present sample. Higher sum scores indicate more symptoms.

We chose 18 putative confounders that covered sociodemographic, environmental, individual, mental health, and diagnostic domains (appendix pp 7, 8) based on a previous association with mental health service contact, or depression. For example, family structure, functioning and mental health problems, peer support, maltreatment, stressful events, socioeconomic status, gender, past referrals for mental health problems, current diagnosis type, severity, and comorbidity have all been related to current mental health service contact.^21^ We assessed seven confounders at multiple timepoints (appendix p 9).

### Statistical analysis

We did primary analyses on an imputed dataset (appendix pp 7, 8) of individuals with complete data for T1 past-year mental health service contact and current mental disorder.

Imputed longitudinal MFQ scores were the outcome in multilevel mixed-effects linear regression models with maximum likelihood estimation, implemented in STATA 13.0. This analysis nests correlated data, thereby accounting for violations in the assumption of independence. For the present data, repeated assessments over time were nested within individuals (the random effect). Fixed effects (ie, predictors in the regression) included linear, quadratic, and categorical effects of age, and confounders (appendix p 7). We assessed categorical effects of T1 disorder and services (unaffected or disorder only or disorder and services) and this variable's interaction with age. We did not include the services-only group of individuals in the primary analysis because without a mental disorder their need for services was less clear. We explored the effects of nesting by school by adding school as a further random-effect.

We did receiver operating characteristic (ROC) analysis to determine the ability of MFQ to classify affective disorder. In ROC analysis the true positive rate (sensitivity) is plotted against the false positive rate (1–specificity). We estimated the area under the curve (AUC) and used it as an index of diagnostic accuracy; a higher AUC reflects greater accuracy. The MFQ has previously been shown to have good-to-high diagnostic accuracy with this method.^13^, ^14^ Additionally, MFQ scores above the 75th percentile are an established behavioural marker for clinical diagnosis of major depression.^22^ The Youdin Index was calculated to determine the clinical cutoff point, because it maximises sensitivity and specificity,^23^ thereby increasing correct classification of individuals with and without depression.

To address the absence of randomisation of mental health service use, a propensity score was generated to weigh the outcome model. A propensity score is the individual probability of attending or receiving a service or treatment conditional on observed baseline covariates. The score is designed to balance confounders between a treatment and control group, as is done in a randomised control trial.^24^ The primary propensity-adjusted analyses comprised data from adolescents with a mental disorder, because those in the disorder-only group were the most appropriate for comparison with the disorder-and-services group (appendix pp 7, 8 provide further details of propensity score). To reduce estimate bias, we first did analyses of the full sample with a disorder, then we restricted the sample to the region of common support—the range of propensity scores which were observed in both treated and untreated individuals.^25^ We estimated the propensity score weighted outcome models with generalised linear modelling (GLM) with a logit link, with adjustment for post-baseline confounding variables. A robust estimator accounted for the sample weighting.

To address the importance of use of a clearly defined need for mental health services based on the presence of a mental disorder, we reanalysed data including all service users, irrespective of disorder.

### Role of the funding source

The funder of the study had no role in study design, data collection, data analysis, data interpretation, or writing of the report. The corresponding author had full access to all the data in the study and had final responsibility for the decision to submit for publication.

## Results

Of the 1238 participants recruited, 1190 adolescents had data for T1 current mental disorder and past-year mental health service contact (appendix p 6). The number of respondents with complete data for all outcomes and covariates at all timepoints was 995 (84%) for T1, 778 (65%) for T2, and 806 (68%) for T3. 64 (5%) adolescents made past-year contact with mental health services; 126 (11%) had a current mental disorder. Among individuals with a disorder, 48 (38%) reported past-year service contact and 46 (96%) of these contacts were based on T1 past-year recall; 36 (84%) of 43 of these adolescents attended five or more sessions (n=5 had missing data for treatment length). In the disorder-and-services group (n=48), disorders were affective (n=16 [33%]), anxiety (n=10 [21%]), behavioural (n=25 [52%]), and other (n=5 [10%]); 14 (29%) of these participants had a comorbid K-SADS diagnosis (appendix p 9).

Overall, 16 (25%) of 64 service users had no disorder, and differed from the disorder-and-services group: baseline MFQ scores were lower in the no-disorder group, although with no significant difference between groups (coefficient −7·64, 95% CI −15·30 to 0·02; p=0·051), and MFQ scores did not change over time (coefficient 1·22, −1·01 to 3·44; p=0·28). Adolescents with a disorder predominantly accessed CAMHS, whereas unaffected adolescents mostly accessed a school counsellor (appendix p 11). Unaffected service users were less antisocial than service users with a disorder (coefficient −3·20, 95% CI 1·10 to 5·29; p=0·0034); remaining covariates p>0·062 (means in appendix p 9).

Adolescents with a disorder were substantially more impaired than unaffected adolescents across all domains of confounders (appendix p 9). When we compared adolescents with a disorder by mental health service contact, individuals varied mainly in diagnostic factors (appendix p 9).

1002 (84%) of 1190 service contacts were reported by both adolescents and caregivers, showing 98% agreement and high chance-corrected agreement (κ=0·78, 95% CI 0·71–0·84). The remaining service contacts were based on either adolescent or caregiver report.

Findings from adjusted multilevel mixed-effects regression analysis revealed that at T1, individuals in both the disorder only and disorder-and-services groups had significantly higher MFQ scores than did those in the unaffected group, but scores between the disorder only and disorder-and-services groups did not differ significantly (table 1, figure). MFQ scores in both these groups improved over time compared with the unaffected group, in which scores remained stable; however, scores improved more quickly among the disorder-and-services group than the disorder-only group (table 1, figure). By T3, scores in the disorder-and-services group had improved (reported reduced symptoms) to the levels of those in the unaffected group (table 1, figure). By contrast, at T3, patients in the disorder-only group reported significantly more symptoms than did those in both the disorder-and-services group and the unaffected group (table 1). Analyses repeated on complete case data yielded similar results (table 1; appendix p 12 shows imputed and complete-case analysis results from unadjusted models). Nesting by school did not affect complete-case results; thus, we did not do clustering during imputation. All data we present for comparability are non-nested results.

ROC analysis revealed MFQ as an excellent discriminator of affective disorder (AUC=0·93, 95% CI 0·90–0·96). The Youden Index indicated an MFQ clinical cutoff point of 22, with 94% sensitivity and 79% specificity, greater than previously obtained in a similar sample measured with differing cutoff point methodology.^14^

We included nine baseline covariates in the propensity score weighting (table 2). Propensity score weighted GLM revealed that among adolescents with a mental disorder, those without contact with mental health services at T1 had nearly four times the odds of being depressed by T3 compared with those in the disorder-and-services group (table 2). Inclusion of post-baseline confounding variables increased odds by more than five times, and in the common support sample, to more than seven times (table 2). Data for propensity score covariates were missing for five (4%) of 124 adolescents with a disorder. To assess the effect of MFQ imputation and missing covariate data on findings, we did unweighted GLM with mental health service contact at T1 predicting T3 MFQ clinical cutoff (adjusted by T1 MFQ only) in three separate models: model A (raw MFQ [n=95]), model B (imputed MFQ [n=124]), and model C (imputed MFQ with missing data from propensity score weighted covariates [n=119]). Effect sizes (calculated from odds ratios^26^) for mental health service contact in these models were similar (0·44 for model A, 0·46 for model B, and 0·45 for model C), indicating no effect of imputation or missing data.

We repeated analyses by expanding the treatment group to include all adolescents who had made past-year contact with mental health services at T1, including 16 individuals with no T1 mental disorder. Comparison groups remained the same as before. The multilevel mixed-effects regression required the same confounding variables as the primary analyses, yielding equivalent results for the unaffected group compared with the other groups. Although this treatment group had the equivalent T1 MFQ to the disorder-only group (coefficient −0·94, 95% CI −3·81 to 1·93; p=0·52) as in the primary analyses, the two groups did not differ in their rate of change over time (linear coefficient −0·68, −2·07 to 0·70; p=0·33; quadratic coefficient −0·27, −0·74 to 0·20; p=0·26). Results did not differ significantly with propensity score weighted GLMs (table 2, appendix pp 7, 8).

## Discussion

To our knowledge, this study is the first in adolescents to support the role of contact with mental health services in improving mental health by late adolescence, while addressing non-randomisation and attrition. Four similar studies^9^, ^10^, ^11^, ^12^ did not address these issues; only one study^9^ adequately controlled for confounding variables, and one other study^10^ showed significant effects that were clinically relevant. Two studies^11^, ^12^ only assessed specialist mental health services, and one study^12^ reported effects of services only if eight or more sessions were attended. In the present study, we considered mental health services from all sectors irrespective of treatment length, we multiply imputed missing data, used propensity score weighting to adjust for participants' initial likelihood to access services, and data yielded clinically relevant results robust to a wide range of confounds. Contact with mental health services appeared to be of such value that after 3 years the levels of depressive symptoms of service users with a mental disorder were similar to those of unaffected individuals. Among adolescents with a mental disorder at age 14 years, the odds of those without past-year contact with mental health services having clinical depression by age 17 years were more than seven times greater than for service users who had been similarly depressed at baseline. Recruitment of participants from the general population, who vary in diagnosis type, severity, and treatment type, and the absence of strict inclusion criteria as in randomised controlled trials also increases the external validity of our study, especially for public mental health and policy makers in the field of community and specialised youth services.

Our findings are in contrast with the null^8^ or negative^7^ association of mental health services reported with longitudinal total emotional and behavioural problems, with no diagnostic threshold. These studies defined mental health services in a similar manner to the present study; one study^7^ implemented propensity matching to address the absence of randomisation. However, measurement of total problems irrespective of clinical typology might mask potential influences of mental health services on emotional or internalising symptoms. Previous null findings can also be explained by a disregard to service need. The present study's findings became non-significant when all users of mental health services were included in the treatment group irrespective of disorder. This outcome underscores the importance of assessment, and supports training of service referrers (eg, in primary care or schools) in the presenting features of mental disorders, to increase the proportion of referrals of adolescents with a clear need who could be more responsive to treatment. Our findings suggest that adolescents accessing mental health services without a mental disorder might be less antisocial, but with fewer symptoms they could be less likely to improve from treatment. Future work should further elucidate this group.

Our study has some limitations. First, verification of the self-report of mental health service use against medical records would have been beneficial; however, findings are supported by high caregiver–adolescent agreement and similar proportions reported in comparable studies in other countries—eg, in adolescents with a DSM-IV diagnosis, 34–56% had past-year contact with any mental health services and 19–25% had contact with specialist mental health services (appendix p 1); the proportions in our study were 38% and 22%, respectively (appendix p 11). Second, heterogeneous treatment makes speculation about a mechanism for improvement difficult. However, common features across treatments could have a role; for example, listening, advice giving, problem solving, being non-judgmental, and being supportive. Larger studies assessing service use separately by treatment type might reveal relative effectiveness, to aid policy makers in determining which services to support. Third, sample size prohibited a focus on participants with a depressive diagnosis; thus, we included adolescents with any DSM diagnosis. However, because adolescents without depression are less likely to show change in depression related to service contact, inclusion of all diagnoses biases the findings to the null. Furthermore, because of numbers of participants, we could not do analyses by varying treatment lengths. However, the intention-to-treat assumption also biases findings to the null; therefore, it is noteworthy that an effect of service use was found. Finally, although addition of covariates and propensity score weighting helped us to address confounding variables, our study had no pretreatment baseline. A larger study with more longitudinal assessments could allow analysis of adolescents initiating service use in a naturalistic setting.^12^

Although our findings are an encouragement to policy makers and commissioners that CAMHS helps to improve mental health, such findings cannot be cause for complacency. Figures published in 2015 show that National Health Service (NHS) spending on children's mental health services in the UK has fallen by 5·4% in real terms since 2010 (£41 million), despite an increase in demand.^27^ Audits have shown a resultant increase in referrals and waiting times; providers report increasingly complex and severe presenting problems, associated with longer stays in inpatient facilities.^28^ The present study occurred in a cohort before these NHS cuts, illustrating to UK policy makers the importance of increasing availability of CAMHS to at least the 2010 levels. Globally, in high-income countries, total mental health spending represents no more than 6% of governmental health expenditures; in many other countries, this figure is less than 1%,^29^ despite mental disorders being one of the leading causes of non-communicable disease burden worldwide.^30^ More studies assessing the effectiveness of CAMHS are needed^28^ for children's mental health to compete for government funds.

When mental health services are ramped-up, care needs to be taken to reach individuals with mental health needs who would typically not access services, comprising more than 60% of those with a mental disorder in our sample. This approach could include increasing community-based services, and ensuring a clear access point to mental health services, such as a designated individual in every school and primary care practice.^28^ Focused training of such individuals in identification of mental disorders could help to prioritise access to mental health services for young people with a clearly defined need, to the betterment of their mental health and wellbeing.

## Figures and Tables

**Figure:**
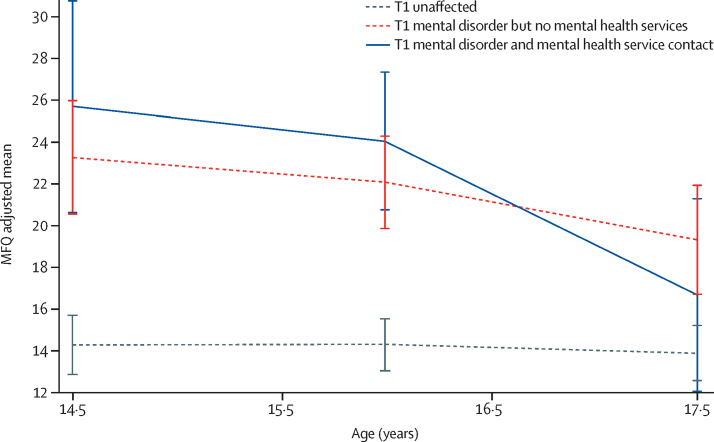
Adolescent MFQ scores by T1 current mental disorder and past-year contact with mental health services Disorder and services variable; imputed and adjusted results. Error bars represent SDs. Adjustments made as for table 1. MFQ=Mood and Feelings Questionnaire. T1=timepoint 1 (age 14·5 years).

**Table 1:** Longitudinal change in MFQ by current mental disorder and past-year contact with mental health services at T1

			**Imputed sample**			**Complete case sample**		
			n	Coefficient (95% CI)	p value	n	Coefficient (95% CI)	p value
**MFQ all timepoints**								
Main effects								
	Disorder and services variable		3302	1·10 (0·47 to 1·72)	0·0011	2469	1·56 (0·95 to 2·17)	<0·0001
	Age (linear)		3302	−0·11 (−0·34 to 0·12)	0·34	2469	−0·24 (−0·45 to −0·02)	0·032
	Age^2^ (quadratic)		3302	0·05 (−0·23 to 0·34)	0·72	2469	−0·36 (−0·62 to −0·10)	0·0075
Disorder and services variable × age								
	Unaffected *vs* disorder only		3302	−1·00 (−1·92 to −0·08)	0·034	2469	−0·34 (−1·25 to 0·57)	0·46
	Unaffected *vs* disorder and services		3302	−2·68 (−3·96 to −1·40)	<0·0001	2469	−2·89 (−4·12 to −1·66)	<0·0001
	Disorder only *vs* disorder and services		3302	−1·68 (−3·22 to −0·14)	0·033	2469	−2·54 (−4·04 to −1·04)	<0·0001
Disorder and services variable × age^2^								
	Unaffected *vs* disorder only		3302	−0·28 (−0·58 to 0·027)	0·074	2469	−0·08 (−0·38 to 0·22)	0·60
	Unaffected *vs* disorder and services		3302	−0·83 (−1·26 to −0·41)	<0·0001	2469	−0·99 (−1·39 to −0·58)	<0·0001
	Disorder only *vs* disorder and services		3302	−0·56 (−1·08 to −0·03)	0·037	2469	−0·91 (−1·40 to −0·41)	<0·0001
Categorical analysis of age								
	Unaffected							
		T1–2	2965	0·02 (−0·70 to 0·74)	0·96	2257	0·59 (−0·07 to 1·24)	0·078
		T2–3	2965	0·22 (−0·52 to 0·96)	0·56	2257	−0·95 (−1·63 to −0·27)	0·0063
		T1–3	2965	0·24 (−0·49 to 0·96)	0·52	2257	−0·36 (−1·00 to 0·28)	0·27
	Disorder only							
		T1–2	202	−2·38 (−5·64 to 0·88)	0·15	140	−0·90 (−4·11 to 2·30)	0·58
		T2–3	202	−0·81 (−4·32 to 2·69)	0·65	140	−1·42 (−4·75 to 1·89)	0·40
		T1–3	202	−3·19 (−6·44 to 0·05)	0·053	140	−2·32 (−5·53 to 0·88)	0·15
	Disorder and services							
		T1–2	126	−4·07 (−9·12 to 0·98)	0·11	72	−1·29 (−6·76 to 4·21)	0·65
		T2–3	126	−3·55 (−9·30 to 2·20)	0·23	72	−7·85 (−14·55 to −1·15)	0·022
		T1–3	126	−7·62 (−12·82 to −2·42)	0·0037	72	−9·13 (−14·81 to −3·44)	0·0016
**T1 MFQ**								
Unaffected *vs* disorder only			1115	5·56 (3·58 to 7·54)	<0·0001	983	5·03 (2·85 to 7·20)	<0·0001
Unaffected *vs* disorder and services			1115	5·61 (2·95 to 8·27)	<0·0001	983	7·52 (4·63 to 10·42)	<0·0001
Disorder only *vs* disorder and services			1115	−0·05 (−3·23 to 3·13)	0·98	983	2·50 (−0·96 to 5·95)	0·16
**T3 MFQ**								
Unaffected *vs* disorder only			1084	2·80 (0·23 to 5·37)	0·033	769	4·20 (1·73 to 6·67)	<0·0001
Unaffected *vs* disorder and services			1084	−1·94 (−5·41 to 1·53)	0·27	769	−1·20 (−4·67 to 2·27)	0·50
Disorder only *vs* disorder and services			1084	−4·74 (−8·80 to −0·68)	0·022	769	−5·40 (−9·47 to −1·34)	0·0085

Data were adjusted as follows: gender, sociodemographics (ethnic origin, Index of Multiple Deprivation, adolescent living with biological parents), environmental factors (number of stressful life events in the past year, current family dysfunction and friendships, any family-focused adversities by T1), and mental health factors (any past Schedule for Affective Disorders and Schizophrenia for School-Age Children diagnosis, any mental health services after T1, any emotional problems in a family member [past 3 years or present], current antisocial traits). Variables not included were any mental health service referral age 0–13 years (p=0·19 in base model) and pubertal status (not a true confounder as p>0·10 and ρ<0·10 with predictor). MFQ=Mood and Feelings Questionnaire. T1=timepoint 1 (age 14·5 years). T2=timepoint 2 (age 16 years). T3=timepoint 3 (age 17·5 years).

**Table 2:** MFQ clinical cutoff point at T3 predicted by propensity score weighted mental health service contact at T1

	Propensity score weighted only*		**Propensity score weighted and adjusted for post-baseline confounds**		**Post-baseline confounds**
	OR (95% CI)	p value	OR (95% CI)	p value	

**Adolescents with a T1 mental disorder: service contact *vs* none**					
Full propensity score sample (n=119)	3·70 (1·40–9·82)	0·0086	5·23 (1·47–18·63)	0·011	T2 MFQ; T3 family dysfunction, stressful life events
Common support sample (n=98)	4·36 (1·41–13·47)	0·011	7·38 (1·73–31·50)	0·0069	T2 MFQ; T3 stressful life events, family dysfunction, living with biological parents
**All with T1 mental health service contact *vs* T1 mental disorder but no services**					
Full propensity score sample (n=134)	1·78 (0·81–3·92)	0·15	2·41 (0·92–6·32)	0·073	T1 MFQ;† mental health service contact after T1; T2 friendships; T3 stressful life events, living with biological parents
Common support sample (n=94)	2·36 (0·93–6·02)	0·072	2·65 (0·88–7·97)	0·085	T1 MFQ; mental health service contact after T1; T3 stressful life events, living with biological parents, family dysfunction

OR=odds ratio. T1=timepoint 1 (age 14·5 years). T2=timepoint 2 (age 16 years). MFQ=Mood and Feelings Questionnaire. T3=timepoint 3 (age 17·5 years).

* Variables used in the propensity score model are ethnic origin, gender, pubertal status, mental health referrals aged 0–13 years, past Schedule for Affective Disorders and Schizophrenia for School-Age Children diagnosis, current behavioural diagnosis, and environmental factors (current friendships and family dysfunction, past-year stressful life events).

† T1 MFQ was used if more strongly related to predictor and outcome than T2 MFQ.
