# Corrections

**DOI:** 10.1016/S2215-0366(17)30132-3

**Published:** 2017-09

*Neufeld SAS, Dunn VJ, Jones PB, Croudace TJ, Goodyer IM. Reduction in adolescent depression after contact with mental health services: a longitudinal cohort study in the UK.* Lancet Psychiatry *2017; **4:** 120–27*—In the summary of this Article, for clarity, the final sentence of the Findings should read “By age 17 years, the odds of reporting clinical depression were higher in individuals without contact than in service users who had been similarly depressed at baseline (adjusted odds ratio 7·38, 1·73–31·50; p=0·0069).” In table 1, data for the imputed sample columns have been updated to reflect changes to the numbers of participants, which are now 3360 for MFQ all timepoints, 3015 for categorical analysis of age for the unaffected subgroup, 213 for the disorder only subgroup, 132 for the disorder and services subgroup, and 1120 for T1 MFQ and T3 MFQ. In the second sentence of the Results, the data should be 983 (83%) for T1, 717 (60%) for T2, and 769 (65%) for T3. In the Discussion, the sixth sentence should read “Among adolescents with a mental disorder at age 14 years, the odds of those without past-year contact with mental health services having clinical depression by age 17 years were greater than for service users who had been similarly depressed at baseline.” These corrections have been made to the online version as of March 27, 2017.

