# Supplementary material for: Reduction in adolescent depression after contact with mental health services: a longitudinal cohort study in the UK
Source: Lancet Psychiatry. 2017 Feb;4(2):120–7. doi: 10.1016/S2215-0366(17)30002-0 (PMC5285445; doi:10.1016/S2215-0366(17)30002-0)
Supplement: Supplementary appendix [file mmc1.pdf]

THE LANCET
Psychiatry

Supplementary appendix

This appendix formed part of the original submission and has been peer reviewed. We post it as supplied by the authors.

Supplement to: Neufeld SAS, Dunn VJ, Jones PB, Croudace TJ, Goodyer IM. Reduction in adolescent depression after contact with mental health services: a longitudinal cohort study in the UK. *Lancet Psychiatry* 2017; published online Jan 10. http://dx.doi.org/10.1016/S2215-0366(17)30002-0.

SUPPLEMENTARY MATERIALS

SUPPLEMENTARY TABLE 1: Prevalence of mental disorder (DSM-IV) in adolescents and past-year mental health service use rates among those with a mental disorder

**Prevalence of mental disorder**										**Past-year mental health service use for those with a mental disorder**						
**Study**	**Data collection**	**Publication**	**Country**	**Diagnostic Instrument**	**Time-frame**	**Informant for diagnosis** [v]	**N**	**Age**	**Prevalence of mental disorder**	**Publication**	**Mental health services definition: type reported**	**Informant for mental health services** [v]	**N**	**Age**	**General Service use rate**	**Specialist mental health services only**
British Child & Adolescent Mental Health Survey (BCAMHS)	1999	Ford et al 2003 [1]	UK	DAWBA	current	a, p, & t (subset)	2,624	13-15	12.2	Meltzer et al, 2000 [2]	Health, social, school [x], alternative	p & t (school services)	992	5-15	71 [z]	---
	2000	Canino et al, 2004 [3]	Puerto Rico	DISC-IV (Spanish)	12 months	a & p	1,897	4-17	16.4	Canino et al, 2004 [3]	SACA (Spanish) – Health, social, school, informal, alternative	a & p	304	4-17	35.5 [y]	20.7 [y]
BCAMHS	2004	Green et al, 2005 [4]	UK	DAWBA	current	a, p, & t (subset)	4,051	11-16	11.5	Green et al, 2005 [4]	Health, social, school [x]	p & t (school services)	700	5-15	70.6 [z]	24.7
National Health & Nutrition Examination Survey (NHANES)	2001-2004	Merikangas et al, 2010 [5]	USA	DISC-IV	12 months	a &/or p depending on disorder	1,894	12-15	24.9 [w]	Merikangas et al, 2010 [5]	Seen someone at hospital, clinic, office	a &/or p depending on disorder	366	8-15	50.6	---
National Comorbidity Survey Adolescent Supplement (NCS-A)	2001-2004	Kessler et al, 2012 [6]	USA	CIDI	current	a & p (subset)	10,148	13-17	23.4	Costello et al, 2014 [7]	SACA	a & p	2,375	13-17	45	22.8
Israel Survey of Mental Health Among Adolescents (ISMEHA)	2004–2005	Farbstein et al, 2010 [8]	Israel	DAWBA (Hebrew)	current	a & p	957	14-17	11.7	Mansbach-Kleinfeld et al, 2010 [9]	A: school [x] (including friends) P: Health, social, school [x], informal, alternative	a & p	a=105 p=110	14-17	a=34 [z] p=40 [z]	---
	2007-2009	Vicente et al, 2012 [10]	Chile	DISC-IV (Spanish)	12 months	a	734	12-18	16.5	Vicente et al, 2012 [10]	SACA (Spanish)	p: 4-11 yrs a: 12-18 yrs	329	4-18	41.6	19.1
	2010-2011	Paula et al, 2014 [11]	Brazil	K-SADS-PL	12 months	p	1721	6-16	13.1	Paula et al, 2014 [11]	Specialist mental health services only	p	226	6-16	---	19.8
Young Minds Matter	2013-2014	Johnson et al, 2016 [12]	Australia	DISC-IV	12 months	a (subset) & p	2442	13-17	19.7	Johnson et al, 2016 [12]	Health, school, informal	a & p	481	13-17	a=44.7 p=56.0	---

[v] a=adolescent; p=parent/caregiver; t=teacher
[w] pooled from Table 1 [5]
[x] includes consulting a class teacher regarding mental health problems as a mental health service contact
[y] pooled from Table 4 [3]
[z] pooled from Table 9.4 [4]

References:

1. Ford T, Goodman R, Meltzer H. The British Child and Adolescent Mental Health Survey 1999: the prevalence of DSM-IV disorders. *J Am Acad Child Adolesc Psychiatry*. 2003;42:1203–11.
2. Meltzer H, Gatward R, Goodman R, Ford T. Mental health of children and adolescents in Great Britain. London: Office for National Statistics; 2000.
3. Canino G, Shrout PE, Rubio-Stipec M, Bird HR, Bravo M, Ramırez R, et al. The DSM-IV rates of child and adolescent disorders in Puerto Rico. *Arch Gen Psychiatry*. 2004;61:85–93.
4. Green H, McGinnity A, Meltzer H, Ford T, Goodman R. Mental health of children and young people in Great Britain, 2004. Basingstoke: Office for National Statistics; 2005.
5. Merikangas KR, He J-P, Brody D, Fisher PW, Bourdon K, Koretz DS. Prevalence and treatment of mental disorders among US children in the 2001-2004 NHANES. *Pediatrics*. 2010;125:75–81.
6. Kessler RC, Avenevoli S, Costello EJ, Georgiades K, Green JG, Gruber MJ, et al. Prevalence, persistence, and sociodemographic correlates of DSM-IV disorders in the National Comorbidity Survey Replication Adolescent Supplement. *Arch Gen Psychiatry*. 2012;69:372–80.
7. Costello EJ, He J-P, Sampson NA, Kessler RC, Merikangas KR. Services for adolescents with psychiatric disorders: 12-month data from the national comorbidity survey-adolescent. *Psychiatr Serv*. 2014;65:359–66.
8. Farbstein I, Mansbach-Kleinfeld I, Levinson D, Goodman R, Levav I, Vograft I, et al. Prevalence and correlates of mental disorders in Israeli adolescents: results from a national mental health survey. *J Child Psychol Psychiatry*. 2010;51:630–9.
9. Mansbach-Kleinfeld I, Farbstein I, Levinson D, Apter A, Erhard R, Palti H, et al. Service use for mental disorders and unmet need: results from the Israel Survey on Mental Health Among Adolescents. *Psychiatr Serv*. 2010;61:241–9.
10. Vicente B, Saldivia S, de la Barra F, Kohn R, Pihan R, Valdivia M, et al. Prevalence of child and adolescent mental disorders in Chile: a community epidemiological study. J Child *Psychol Psychiatry*. 2012;53:1026–35.
11. Paula CS, Bordin IAS, Mari JJ, Velasque L, Rohde LA, Coutinho ESF. The mental health care gap among children and adolescents: data from an epidemiological survey from four Brazilian regions. *PLoS One*. 2014;9:e88241.
12. Johnson SE, Lawrence D, Hafekost J, Saw S, Buckingham WJ, Sawyer M, et al. Service use by Australian children for emotional and behavioural problems: Findings from the second Australian Child and Adolescent Survey of Mental Health and Wellbeing. *Aust New Zeal J Psychiatry*. 2016: DOI:10.1177/0004867415622562.

SUPPLEMENT 1

Measures used to obtain mental health services information

Note: all data was coded / recoded from the measures below by SASN, in consultation with VJD, to conform to variables on Supplementary Table 2.

(I) THE CAMBRIDGE EARLY EXPERIENCE INTERVIEW (CAMEEI) version 2, January 2012 by Valerie J Dunn & Ian M Goodyer. A research interview with parents/primary caregivers to assess their child's exposure to family-focused adversities through childhood and adolescence, obtained at T1 caregiver interview. Below are the questions relevant to mental health services. Core questions, asked verbatim, are in bold and these are followed by researcher-led discussions based on sets of prompting questions.

Proband psychiatric problems

Code for each time period (ages 0-5, 5-11, 11-14). This may be a suitable time to ask consent to confirm details in clinical notes. Do not record minor illness/injuries.

- Has … ever suffered any emotional, behavioural or other problems? *Follow questions below for specific problems.*

Specific disorders:

-Has s/he ever seemed very low for weeks on end? –Been unable to enjoy things?
-As far as you know has s/he ever hurt themselves on purpose or attempted suicide?

-Is/was s/he a worrier? What about? -Has … ever had a panic attack?
-Ever been very frightened of a specific thing?
-Ever had to check things repeatedly, or do things over and again?
-Been a serious worrier? About what? How bad does/did it get?

-Strictly dieted/lost weight? Had regular eating binges? Deliberately sick after food?

-What about attention or concentration problems? -Does s/he act impulsively?
-Can s/he sit still when needed?

-Been in trouble with police? -Got into a lot of fights or been violent?
-Been accused of bullying others? -Excluded from school?
-Loses his/her temper a lot? -Argues with adults and won't do as told?
-Have you been concerned about his/her drinking or taking drugs?

-Was …. referred to see a clinician for any of these problems, like a psychiatrist, counsellor or similar person? *Note details.*
-Did you/they take up the referral? What was the diagnosis/outcome/treatment (if any)?

Then establish:
-Proband age/s of onset? -Referred? Taken up? -Treatment?
-Full recovery? Duration of illness?

**Person affected:** PFC, PMC, sib, proband				
**Diagnosis**:	1=affective	2=anxiety	3=ADHD/ADD	4=CD/ODD
	5=substance/alco	6=NSSI	7=other	
**Referred to**:	1=GP	2=mental health service	3=Ed. psych	
	4=family therapy	5=counsellor	6=other	
**Taken up:**	0=No	1=Yes		
**Age/s of proband at onset:**	**If T3, current:** 0=no 1=yes			
**Treatment:**	0=none	1=GP		
	2=single psych o'pat	3=regular psych outpatient		
	4=admission	5=other		
**Full recovery:**	0=no, 1=yes	**Duration/s** (weeks):		

(II) FAMILY AND FAMILY HEALTH (parent self-report questionnaire T1 [age 14.5] and T3 [age 17.5], developed by Ian M Goodyer for the ROOTS study)

HEALTH OF ROOTS TEENAGER:

(Following questions regarding any emotional/nervous illness and behaviour problems in their son/daughter…)

Has your son/daughter **ever** been referred to a psychiatrist, educational psychologist or similar person? **IF YES**, please give details (*who to, why, when, treatment):* **Yes No**

____
____
____

(III) KESSLER PSYCHOLOGICAL DISTRESS SCALE (K10)
http://www.hcp.med.harvard.edu/ncs/ftpdir/k6/K10+self%20admin-3-05-%20FINAL.pdf

Source: Kessler R. Professor of Health Care Policy, Harvard Medical School, Boston, USA.
(T3 proband self-report questionnaire)

These questions are about how you have been feeling during the **PAST MONTH**.

For each question, please circle the number that best describes how often you had this feeling.

Q1

**During that month, how often did you feel ….**	**All of the time**	**Most of the time**	**Some of the time**	**A little of the time**	**None of the time**
**a**…tired out for no good reason	1	2	3	4	5
**b**...nervous?	1	2	3	4	5
**c**…so nervous that nothing could calm you down	1	2	3	4	5
**d**…hopeless?	1	2	3	4	5
**e**…restless or fidgety?	1	2	3	4	5
**f**…so restless that you could not sit still?	1	2	3	4	5
**g**…depressed?	1	2	3	4	5
**h**…so depressed that nothing could cheer you up?	1	2	3	4	5
**i**...that everything was an effort?	1	2	3	4	5
**j**…worthless?	1	2	3	4	5

Q5 During the past month, how many times did you see a doctor or other health professional about these feelings?

__________ **(Number of days)**

(IV) TREATMENT AND REFERRAL HISTORY (T3 [age 14.5] adolescent interview, developed by Ian Goodyer for the ROOTS study)

Has anyone ever suggested you see, or referred you to, someone like a counsellor, psychologist or psychiatrist for any concerns you or they have about your mood or behaviour or any worries about something that's happened to you?

**Presenting Problem:**			
**Date of onset:**			
**Referral?**	Yes/No	Yes/No	Yes/No
**Referred: (circle)**	1.GP<br>2.Counsellor<br>3.Family Therapy<br>4.Education psych<br>5.Psychiatrist<br>6.Other:________________<br>7. Referred, but unknown	1.GP<br>2.Counsellor<br>3.Family Therapy<br>4.Education psych<br>5.Psychiatrist<br>6.Other:________________<br>7. Referred, but unknown	1.GP<br>2.Counsellor<br>3.Family Therapy<br>4.Education psych<br>5.Psychiatrist<br>6.Other:________________<br>7. Referred, but unknown
**Treatment: (circle)**	1.None<br>2.Single outpatient or assessment<br>3. Regular outpatient<br>4. Admitted<br>5. Other:_______________	1.None<br>2.Single outpatient or assessment<br>3. Regular outpatient<br>4. Admitted<br>5. Other:_______________	1.None<br>2.Single outpatient or assessment<br>3. Regular outpatient<br>4. Admitted<br>5. Other:_______________
**Adherence to treatment**	Yes/No	Yes/No	Yes/No
**Duration of Treatment**			
**Medication**			
**Duration of meds:**			
**Adherence to meds?**			
**Notes:**			

SUPPLEMENTARY FIGURE 1: Flowchart of participants at T1 (age 14·5) with data on mental health service contact and mental disorder. MFQ=Mood and Feelings Questionnaire.

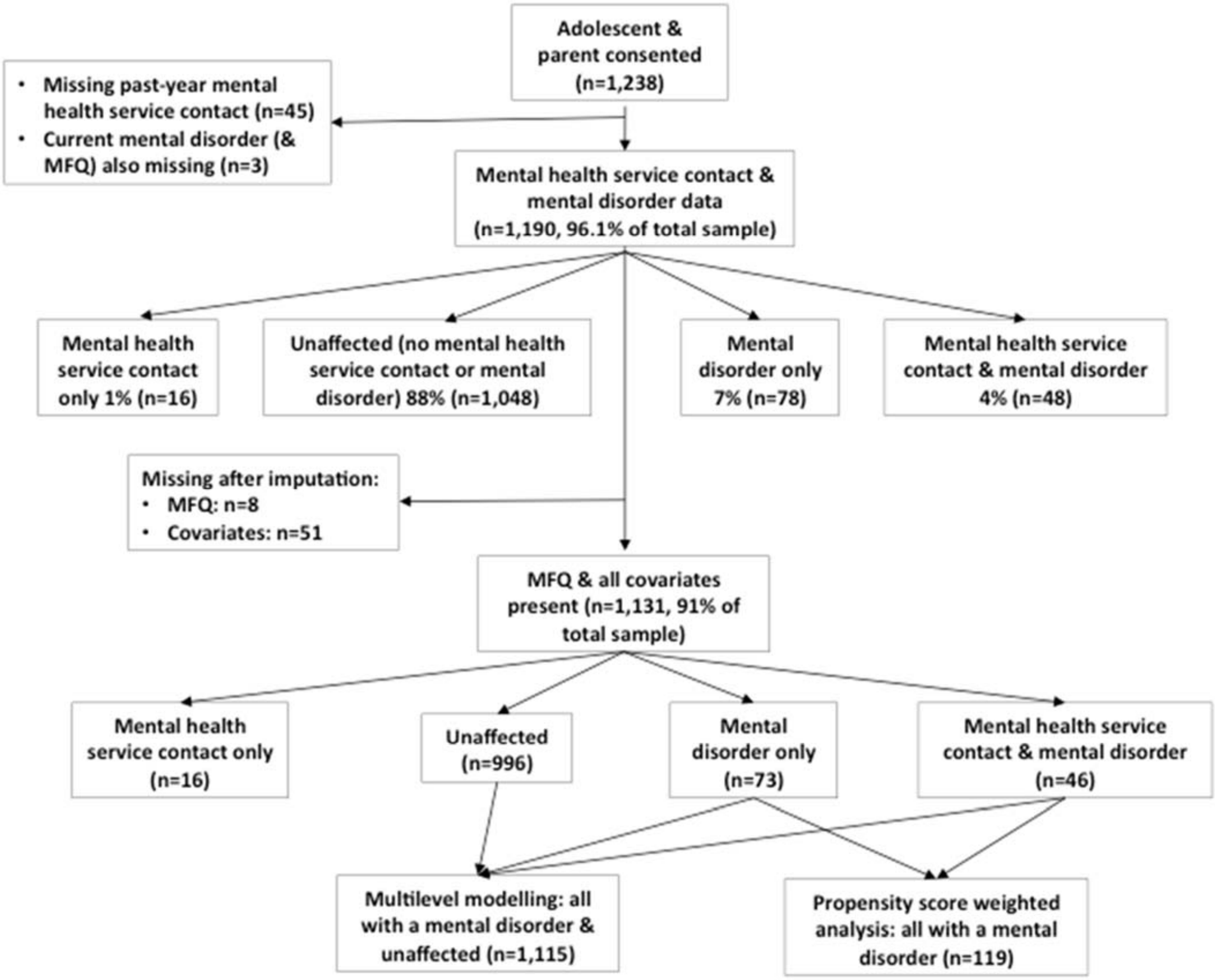

SUPPLEMENT 2: Additional method and results details

Method

Putative confounders: (Supplementary Table 2 contains data source and time-point when data was obtained.)
Socio-demograhics: ethnicity, Index of Multiple Deprivation[1] (based on baseline post code), whether the adolescent was living with his/her biological parents.
Environmental factors: number of stressful life events in the past year (Life Events Questionnaire[2]), current family dysfunction (McMaster Family Assessment Device[3]) and friendships (Cambridge Friendship Scale[4]), any family-focused adversities by T1 (age 14.5; Cambridge Early Experiences Interview[5]).
Individual factors: gender, pubertal status (Tanner stages[6]).
Mental Health factors: any past K-SADS diagnosis, any mental health service referral age 0-13, any mental health services after T1, any emotional problems in a family member (past 3 years or present), current antisocial traits (Antisocial Process Screening Device[7]).
Diagnostic factors (those with a T1 mental health disorder only): diagnosis type, severity (based on Children's Global Assessment Scale[8]: mild (60-51), moderate (50-41), or severe (40-31)), and presence of comorbidity.
Total scores were used for continuous measures if at least 85% of items were completed, or 100% for measures containing 14 or fewer items.

Multiple Imputation:
For longitudinal measures which were continuous sum scores (MFQ, friendships, antisocial traits, and family dysfunction), missing data from all three time-points were imputed separately by measure, with each model consisting of all items from the measure at all time-points, as well as gender, socio-economic status, and DSM diagnosis at T1 (yes/no), related to attrition throughout the study. Following item imputation, measures were re-scored based on criteria above. Next, categorical and ordinal variables obtained at T1 and T3 (age 17.5; any emotional problems in a family member, adolescent living with biological parents, number of stressful life events in the past year) were imputed along with baseline-only categorical variables (ethnicity, socio-economic status, pubertal status, and family-focused adversities). Also included in this imputation model were an additional 13 variables which were used in the outcome models or predicted missingness (available from the first author upon request). Using the ice command in Stata,[9] twenty chained equations were created, a greater number than the percentage of missing outcome data[10]. This method assumes data are missing at random, a reasonable assumption given the ability of many variables to predict missingness. Rubin's rules were used when combining the imputed datasets for analysis[11].

Change in Depression Scores:
In order to control for confounding, baseline covariates related to both the predictor (T1 disorder and services variable) and outcome (T3 MFQ) $p<0{\cdot}10$ or Pearson' r or $\rho=>0{\cdot}10$ were individually put in a multi-level model of T1 disorder and services predicting MFQ across time (base model), with non-significant covariates excluded from full models. Diagnostic factors were not included as covariates, as by definition they did not apply to the control group, and were thus collinear with the predictor ($\rho>0{\cdot}84$). Any models which involved post-baseline MFQ also controlled for any service usage after baseline. All twelve covariates which correlated $p<0{\cdot}10$ with both T3 MFQ and T1 disorder and services (Supplementary Table 2b) remained in the full model, having retained $p<0{\cdot}10$ in separate base models, except mental health referrals age 0-13 ($p=0{\cdot}18$; covariate inter-correlations$<0{\cdot}50$).

Propensity Score Adjusted Analyses:
Similar to the present study, propensity scoring has been used to adjust for confounds in a birth cohort investigating whether reported psychotropic drug use was associated with improvement in depressive symptoms.[12] In the present study, the propensity score was estimated using logistic regression, with baseline covariates correlated to the outcome (MFQ clinical cut-off age 17 $>=0{\cdot}10$) used to predict baseline mental health service contact regardless of the covariate relationship with mental health service contact.[13,14] The propensity score method used to check covariate balance between groups and weight the data was inverse probability of treatment weighting (IPTW). IPTW gives correct estimations of treatment effect in small sample sizes,[14] and on average is similar to the treatment effect in randomized studies, unlike other propensity scoring methods.[15] Stabilized IPTWs were used to reduce impact of extreme weights, thus reducing estimate bias.[16] The propensity score adjusted outcome models were estimated with each IPTW as the analytical weight.[17] Post-baseline covariates (including prior MFQ, see Supplementary Table 2b) were included as confounders if correlated $>=0{\cdot}10$ with both the weighted outcome and predictor (calculated separately in full sample and common support sample).

Results

Propensity score weighted models for diagnosed sample:
Unbalanced covariates prior to propensity score weighting are indicated on Supplementary Table 2b. After weighting, mental health referrals age 0-13 and current comorbidity remained unbalanced between those with a current mental disorder who had, and had not accessed mental health services in the past year. Referrals age 0-13 was added to the propensity score model, being more

related to the outcome and less related to the predictor than current comorbidity.[13] Thereafter, all covariates were balanced (standardized differences <0·42, all ns, Supplementary Figure 2), indicating correct specification of the propensity score model.[18]

Propensity score weighted models for all Service Users:
For the propensity score weighted analyses, baseline covariates that correlated with T3 (age 17.5) MFQ cut-off >=0·10 were identical to those in the diagnosed only sample, except past K-SADS diagnosis was not correlated. After weighting, past K-SADS diagnosis, family-focused adversities, referrals age 0-13, and current anxiety diagnosis were unbalanced. These were iteratively added to the propensity score model, except family-focused adversities, which became balanced upon addition of past K-SADS diagnosis. Current comorbidity was then unbalanced; after its addition to the model all covariates were balanced (SDiff<0·24).

References:

1. Noble M, McLennan D, Wilkinson K, Whitworth A, Barnes H, Dibben C. The English Indices of Deprivation 2007. London: Department for Communities and Local Government; 2008.
2. Goodyer IM, Herbert J, Tamplin A, Altham PM. Recent life events, cortisol, dehydroepiandrosterone and the onset of major depression in high-risk adolescents. *Br J Psychiatry*. 2000;177:499–504.
3. Epstein NB, Baldwin LM, Bishop DS. The McMaster Family Assessment Device. J Marital Fam Ther. 1983;9:171–80.
4. Atkin A, Corder K, Goodyer I, Bamber D, Ekelund U, Brage S, et al. Perceived family functioning and friendship quality: cross-sectional associations with physical activity and sedentary behaviours. *Int J Behav Nutr Phys Act*. 2015;12:1–9.
5. Dunn VJ, Abbott RA, Croudace TJ, Wilkinson P, Jones PB, Herbert J, et al. Profiles of family-focused adverse experiences through childhood and early adolescence: The ROOTS project a community investigation of adolescent mental health. *BMC Psychiatry*. 2011;11:1–16.
6. Tanner R. Growth at Adolescence. Oxford: Blackwell; 1962.
7. Frick PJ, Hare RD. The Antisocial Process Screening Device (APSD). Toronto: Multi-Health Systems; 2001.
8. Shaffer D, Gould MS, Brasic J, Ambrosini P, Fisher P, Bird H, et al. A Children's Global Assessment Scale (CGAS). *Arch Gen Psychiatry*. 1983;40:1228–31.
9. Royston P, White I. Multiple Imputation by Chained Equations (MICE): implementation in Stata. *J Stat Softw*. 2011;45:1–20.
10. White I, Royston P, Wood A. Multiple imputation using chained equations: Issues and guidance for practice. *Stat Med*. 2011;30:377–99.
11. Rubin D. Multiple imputation for nonresponse in surveys. Hobokin: John Wiley and Sons; 2004.
12. Colman I, Croudace TJ, Wadsworth MEJ, Kuh D, Jones PB. Psychiatric outcomes 10 years after treatment with antidepressants or anxiolytics. *Br J Psychiatry*. 2008;193:327–31.
13. Brookhart MA, Schneeweiss S, Rothman KJ, Glynn RJ, Avorn J, Stürmer T. Variable selection for propensity score models. *Am J Epidemiol*. 2006;163:1149–56.
14. Pirracchio R, Resche-Rigon M, Chevret S. Evaluation of the propensity score methods for estimating marginal odds ratios in case of small sample size. *BMC Med Res Methodol*. 2012;12:1–10.
15. Ali MS, Groenwold RHH, Belitser S V, Pestman WR, Hoes AW, Roes KCB, et al. Reporting of covariate selection and balance assessment in propensity score analysis is suboptimal: a systematic review. *J Clin Epidemiol*. 2015;68:122–31.
16. Robins JM, Hernan MA, Brumback B. Marginal structural models and causal inference in epidemiology. *Epidemiology*. 2000;11:550–60.
17. Linden A, Adams JL. Evaluating health management programmes over time: Application of propensity score-based weighting to longitudinal data. *J Eval Clin Pract*. 2010;16:180–5.
18. Austin PC. An introduction to propensity score methods for reducing the effects of confounding in observational studies. *Multivariate Behav Res*. 2011;46:399–424.

SUPPLEMENTARY TABLE 2: T1 sample characteristics by T1 mental disorder and mental health service contact.

	**No mental disorder T1 mean (sd) or n (%)**		**Mental disorder T1 mean (sd) or n (%)**		**Total n available with mental disorder and mental health service contact data**	**p value [c]**	
**Characteristics**	**Unaffected: no mental health service contact (n=1,048)**	**Mental health service contact only (n=16)**	**No mental health service contact (n=78)**	**mental health service contact (n=48)**		**Any mental disorder vs. unaffected**	**Mental health service contact (no/yes) in diagnosed**
**Socio-demographic Factors:**							
Index of Multiple Deprivation	8.2 (5.3)	11.2 (5.4)	9.5 (7.2)	9.2 (6.2)	1,187	0.020	0.80
Ethnicity (% White)	953 (93%)	16 (100%)	71 (97%)	44 (98%)	1,154	0.085	0.86
Living with biological parents [b] (%)	733 (72%)	6 (38%)	42 (60%)	26 (59%)	1,149	0.0063	0.92
**Environmental Factors:**							
Family-focused adversities (P; % moderate/severe)	309 (31%)	12 (80%)	33 (45%)	27 (61%)	1,140	<0.0001	0.090
Friendships [a]	25.7 (4.1)	22.2 (5.4)	24.1 (4.7)	23.1 (5.3)	1,134	<0.0001	0.29
Family dysfunction [b]	22.1 (5.5)	22.5 (6.3)	24.3 (6.8)	26.6 (7.4)	1,105	<0.0001	0.12
Stressful life events [b] (% with at least one)	348 (34%)	10 (71%)	34 (48%)	27 (63%)	1,147	<0.0001	0.12
**Individual Factors:**							
Gender (% female)	561 (54%)	11 (69%)	52 (67%)	27 (56%)	1,190	0.051	0.24
Post-pubertal (%)	924 (91%)	15 (94%)	68 (90%)	40 (89%)	1,156	0.61	0.92
**Mental Health Factors:**							
Any mental health service referral, age 0-13 (%)	79 (8%)	8 (50%)	12 (15%)	22 (45%)	1,190	<0.0001	0.00019
Past K-SADS diagnosis (%)	83 (8%)	6 (38%)	14 (18%)	13 (27%)	1,190	<0.0001	0.23
Emotional problems in family member [b] (% past or present, P)	156 (15%)	3 (19%)	20 (28%)	17 (38%)	1,152	<0.0001	0.26
MFQ	14.3 (9.1)	22.9 (9.9)	24.0 (12.3)	26.9 (14.6)	1,160	<0.0001	0.26
Antisocial traits [a] (P)	8.0 (5.1)	8.4 (4.3)	10.5 (6.0)	14.8 (7.9)	1,147	<0.0001	0.0013
**Diagnostic Factors:** (sample with a mental health disorder)							
Affective diagnosis (%)	---	---	15 (19%)	16 (33%)	126	---	0.074
Anxiety diagnosis (%)	---	---	43 (55%)	10 (21%)	126	---	0.00015
Behavioural diagnosis (%)	---	---	19 (24%)	25 (52%)	126	---	0.0015
Other diagnosis (%)	---	---	4 (5%)	5 (10%)	126	---	
Comorbidity (%)	---	---	5 (6%)	14 (29%)	126	---	0.00053
Moderate or severe impairment (%)	---	---	9 (12%)	28 (58%)	126	---	<0.0001

T1=timepoint 1 (age 14.5 years). T2=timepoint 2 (age 16 years). T3=timepoint 3 (age 17.5). P=primary caregiver report (adolescent-report was used unless specified)
[a] assessed T1, T2, and T3; [b] assessed ages T1 and T3; assessed only at T1 unless specified. Additionally, any mental health services after T1 was reported at T3 by primary caregiver and/or adolescent
[c] ANOVAs used for continuous variables, Chi-square tests for categorical variables

SUPPLEMENTARY TABLE 2b: Correlation of T1 covariates with outcome (T3 MFQ) and predictor (T1 disorder and/or service variable), results after imputation

**Covariates (T1 unless specified)**	**T1 mental disorder + unaffected sample[a] (n=1,137-1,166)**		**Sample with a T1 mental disorder (n=118-124)**	
	**T3 MFQ (continuous)**	**T1 disorder and/or service**	**T3 MFQ clinical cut-off**	**T1 mental health service contact**
**Socio-demographic Factors:**				
Index of Multiple Deprivation	0·02	0·09*	0·08	-0·04
Ethnicity (White vs. other)	0·14**	-0·19*	0·27	0·03
Living with biological parents	-0·11*****	-0·14***	-0·07	-0·01
**Environmental Factors:**				
Family-focused adversities (none/mild vs. moderate/severe)	0·10***	0·22******	0·01	0·15
Friendships	-0·27******	-0·22******	-0·15	-0·09
Family dysfunction	0·25******	0·28******	0·18+	0·22* (u)
Stressful Life events	0·18******	0·24******	0·24***	0·13
Any mental health services after T1	0·31******	0·46******	0·11	0·23
**Individual Factors:**				
Gender (0=male; 1=female)	0·24******	0·12+	0·37*	-0·13
Pubertal status (pre- vs post-)	0·12******	-0·03	0·21+	-0·04
**Mental Health Factors:**				
Any mental health service referral age 0-13	0·13**	0·44******	-0·06	0·50*** (u)
Past K-SADS diagnosis	0·17*****	0·34******	-0·25	0·21
Emotional problems in family member (past or present)	0·09***	0·20******	-0·01	0·12
Antisocial Traits	0·07*	0·33******	0·02	0·23*** (u)
**Diagnostic Factors** (diagnoses weighted by severity):				
Affective diagnosis	---	---	0·06	0·34*
Anxiety diagnosis	---	---	-0·01	-0·34** (u)
Behavioural diagnosis	---	---	-0·10	0·49***** (u)
Comorbidity	---	---	0·02	0·51*** (u)

All baseline covariates that correlated with T3 MFQ clinical cut-off>=0·10 were included in the propensity score model. T1=timepoint 1 (age 14.5 years). T2=timepoint 2 (age 16 years). T3=timepoint 3 (age 17.5). MFQ=Mood and Feelings Questionnaire. (u) = baseline covariates unbalanced prior to propensity score weighting..

[a] Unaffected (no mental disorder or mental health services), disorder only, and disorder and services groups

+p<0·1, *p<0·05, **p<0·01 ***p<0·005, **** p<0·001, ***** p<0·0005, ****** p<0·0001

SUPPLEMENTARY TABLE 3: Details of T1 mental health referrals and mental health service contact, n (%)

Mental health referrals and service contact details	Current mental disorder & past year mental health service contact (n=48)	Current mental disorder, no past year mental health service contact (n=78)	No mental disorder but past year mental health service contact (n=16)
No. of mental health referrals [a] from birth to T1			
0	0	61 (78%)	0
1	34 (71%)	15 (19%)	13 (81%)
2	8 (17%)	2 (3%)	3 (19%)
3	6 (12%)	0	0
No. of mental health referrals post T1-T3 (age 17.5)			
0	33 (69%)	54 (69%)	8 (50%)
1	11 (23%)	15 (19%)	5 (31%)
2	4 (8%)	8 (10%)	2 (13%)
3	0	1 (1%)	1 (6%)
Any mental health service contact post T1-T3	24 (50%)	29 (37%)	10 (63%)
Reason for T1 mental health service contact (past year)			
Anger/behaviour	8 (17%)		3 (19%)
Mood/stress problems	12 (25%)		6 (38%)
ADHD	9 (19%)		0
Deliberate Self Harm	4 (8%)		0
Eating problems	2 (4%)		0
Family/relationship problems	2 (4%)		1 (6%)
Trauma	1 (2%)		1 (6%)
Bullied	0		3 (19%)
Bereavement	0		1 (6%)
> 1 reason	10 (21%) [b]		0
Unknown/missing	0		1 (6%)
T1 Referral source [c] (past year)			
GP	17 (36%)		3 (19%)
School	16 (33%)		9 (56%)
Family/self	4 (8%)		2 (13%)
Health Visitor	2 (4%)		0
Other	1 (2%)		0
Unknown/missing	8 (17%)		2 (13%)
T1 mental health service type (past year)			
CAMHS (Child and Adolescent Mental Health services)[d]	26 (54%)		0
School counsellor	8 (17%)		9 (56%)
Charity counsellor	3 (6%)		0
GP	2 (4%)		0
GP counsellor	0		1 (6%)
>1 Sector [e]	5 (10%)		0
Educational psychologist	0		1 (6%)
Counsellor or psychologist unspecified	3(6%)		3 (19%)
Not specified	1 (2%)		2 (13%)
T1 Treatment duration (past year)			
1-3 sessions	7 (15%)		2 (13%)
5 or more sessions	36 (75%)		12 (75%)
Not specified	5 (10%)		2 (13%)

T1=timepoint 1 (age 14.5 years). T2=timepoint 2 (age 16 years). T3=timepoint 3 (age 17.5).

[a] Referrals were considered separate if they were to a different service type, or there was a distinct break in service use

[b] 9 cited mood/stress problems as one of the reasons

[c] for consecutive referrals, the referral source is coded from the first referral

[d] Only one participant had used inpatient services

[e] 2 of these individuals were referred to CAMHS as one of the sectors. Thus, past-year CAMHS referral rates are 22% (28/126) of those with a mental disorder

SUPPLEMENTARY TABLE 4: Longitudinal change in MFQ by current mental disorder and past-year mental health service contact at T1, unadjusted

	Imputed Sample			Complete Case Sample		
**MFQ ALL TIMEPOINTS**	**n**	**Coefficient (95% CI)**	**p**	**n**	**Coefficient (95% CI)**	**p**
**Main Effects:**	3,498			3,008		
Disorder and services variable		3·14 (2·43, 3·85)	<0·0001		3·52 (2·86, 4·18)	<0·0001
Age (linear)		-0·27 (-0·49, -0·05)	0·018		-0·47 (-0·68, -0·26)	<0·0001
Age^2 (quadratic)		-0·20 (-0·50, 0·09)	0·18		0·15 (-0·11, 0·40)	0·27
**Disorder and services variable*age**	3,498			3,008		
Unaffected vs disorder only		-1·41 (-2·31, -0·51)	0·0024		-1·29 (-2·12, -0·45)	0·0025
Unaffected vs disorder and services		-2·96 (-4·16, -1·75)	<0·0001		-2·99 (-4·10, -1·89)	<0·0001
Disorder only vs disorder and services		-1·55 (-3·01, -0·08)	0·038		-1·71 (-3·06, -0·35)	0·013
**Disorder and services variable*age^2**	3,498			3,008		
Unaffected vs disorder only		-0·41 (-0·70, -0·12)	0·0063		-0·41 (-0·68, -0·14)	0·0033
Unaffected vs disorder and services		-0·93 (-1·32, -0·54)	<0·0001		-1·06 (-1·42, -0·70)	<0·0001
Disorder only vs disorder and services		-0·52 (-1·00, -0·04)	0·033		-1·47 (-1·93, -1·00)	<0·0001
**Categorical analysis of age**						
Unaffected :	3,126			2,710		
T1-2		0·31 (-0·45, 1·07)	0·42		-0·83 (-1·48, -0·18)	0·013
T2-3		-0·56 (-1·31, 0·19)	0·14		-0·03 (-0·70, 0·64)	0·94
T1-3		-0·25 (-0·94, 0·44)	0·48		-0·85 (-1·48, -0·23)	0·0077
Disorder only:	234			196		
T1-2		-2·46 (-5·87, 0·96)	0·16		-2·59 (-5·54, 0·36)	0·085
T2-3		-2·01 (-5·38, 1·35)	0·24		-2·06 (-5·08, 0·95)	0·18
T1-3		-4·47 (-7·50, -1·45)	0·0041		-4·66 (-7·46, -1·86)	0·0011
Disorder and services:	138			102		
T1-2		-3·43 (-8·40, 1·54)	0·18		-0·96 (-6·70, 4·78)	0·74
T2-3		-5·69 (-10·69, -0·68)	0·026		-9·06 (-15·08, -3·04)	0·0032
T1-3		-9·12 (-13·88, -4·36)	<0·0001		-10·02 (-14·96, -5·09)	<0·0001
**T1 MFQ**	1,166			1,138		
Unaffected vs disorder only		9·64 (7·37, 11·91)	<0·0001		9·75 (7·49, 12·01)	<0·0001
Unaffected vs disorder and services		12·50 (9·57, 15·44)	<0·0001		12·63 (9·73, 15·52)	<0·0001
Disorder only vs disorder and services		-2·86 (-6·45, 0·73)	0·12		2·87 (-0·70, 6·45)	0·12
**T3 MFQ**	1,166			993		
Unaffected vs disorder only		5·42 (2·81, 8·03)	<0·0001		5·36 (2·82, 7·91)	<0·0001
Unaffected vs disorder and services		3·64 (0·19, 7·08)	0·039		3·82 (0·39, 7·24)	0·030
Disorder only vs disorder and services		1·79 (-2·40, 5·97)	0·40		-1·55 (-5·72, 2·63)	0·47

MFQ=Mood and Feelings Questionnaire. T1=timepoint 1 (age 14.5 years). T2=timepoint 2 (age 16 years). T3=timepoint 3 (age 17.5).

SUPPLEMENTARY FIGURE 2: Standardized differences of baseline covariates between baseline mental health service contact groups in the sample with a mental disorder, pre- and post-stabilised Inverse Probability of Treatment Weighting (IPTW) adjustment.

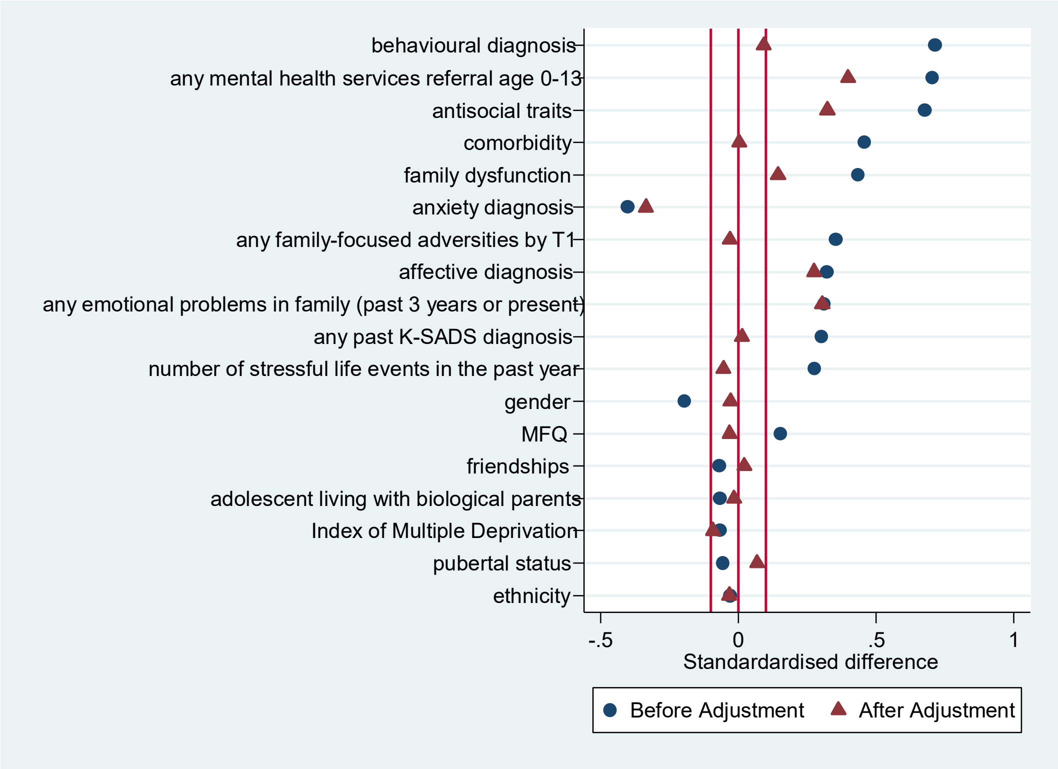

K-SADS=Schedule for Affective Disorders and Schizophrenia for School-Age Children. MFQ=Mood and Feeling Questionnaire. Variables reflect current status unless specified.
